# Zoonotic Alphaviruses in Fatal and Neurologic Infections in Wildlife and Nonequine Domestic Animals, South Africa

**DOI:** 10.3201/eid2606.191179

**Published:** 2020-06

**Authors:** Jumari Steyn, Isabel Fourie, Johan Steyl, June Williams, Voula Stivaktas, Elizabeth Botha, Stefanie van Niekerk, Bjorn Reininghaus, Marietjie Venter

**Affiliations:** University of Pretoria, Pretoria, South Africa (J. Steyn, I. Fourie, J. Steyl, J. Williams, V. Stivaktas, E. Botha, S. van Niekerk, M. Venter);; Mpumalanga Veterinary Services, Middelburg, South Africa (B. Reininghaus)

**Keywords:** Alphaviruses, viruses, Middelburg virus, Sindbis virus, neurologic infections, meningitis/encephalitis, nonequine domestic animals, wildlife, zoonoses, South Africa

## Abstract

Alphaviruses from Africa, such as Middelburg virus (MIDV), and Sindbis virus (SINV), were detected in horses with neurologic disease in South Africa, but their host ranges remain unknown. We investigated the contribution of alphaviruses to neurologic infections and death in wildlife and domestic animals in this country. During 2010–2018, a total of 608 clinical samples from wildlife and nonequine domestic animals that had febrile, neurologic signs or unexplained deaths were tested for alphaviruses. We identified 32 (5.5%) of 608 alphavirus infections (9 SINV and 23 MIDV), mostly in neurotissue of wildlife, domestic animals, and birds. Phylogenetic analysis of the RNA-dependent RNA polymerase gene confirmed either SINV or MIDV. This study implicates MIDV and SINV as potential causes of neurologic disease in wildlife and nonequine domestic species in Africa and suggests a wide host range and pathogenic potential.

Alphaviruses (family *Togaviridae*) have been recognized as major emerging viruses. New World alphaviruses, such as Western equine encephalitis virus, Eastern equine encephalitis virus, and Venezuelan equine encephalitis virus, are traditionally associated with severe disease, such as encephalitis and a high mortality rate in humans and horses in the Americas and Australia (*1*,*2*). Old World alphaviruses, such as o’nyong nyong virus, chikungunya virus, and Sindbis virus (SINV), are associated mostly with arthralgia, although rare infections with neurologic disease have been reported in humans and equids (*3*). Chikungunya virus was responsible for millions of human infections in new territories, and although the case-fatality rate was low, outbreaks resulted in major illness and long-term sequelae in affected persons (*4*,*5*). Old and New World alphaviruses cluster in separate phylogenetic groups (*6*), but limited information is available about pathogenesis and host range of alphaviruses from Africa and pathogenesis in animals.

SINV is a human pathogen that is distributed across Africa, Europe, Australia, and Asia (*7*). Large outbreaks of infection have been recorded in humans in South Africa since 1974 (*8*). Limited studies on the disease potential of SINV in animals have been conducted. The reservoir hosts are primarily migratory birds (*9*,*10*); various *Culex* mosquitoes are the main vectors. Recent reports suggest that SINV might cause febrile and neurologic disease in horses in South Africa (*11*,*12*).

Middelburg virus (MIDV) was discovered in the late 1950s in *Aedes* species mosquitoes (*13*), although it was only linked to disease in 1990 (*14*) after MIDV was isolated from a horse with signs of infection with African horse sickness virus in Zimbabwe (*14*). Since that time, MIDV has been implicated as the etiologic agent of neurologic and febrile disease in horses in South Africa (*11*). Seroprevalence studies in South Africa during 1959–1960 identified MIDV antibodies in humans, cattle, sheep, and goats (*15*,*16*). The vector status of MIDV is largely unknown; only *Ae. caballus* and *Mansonia africana* mosquitoes have been implicated (*17*). Little is known about the epidemiology of MIDV, including confirmed reservoir hosts, susceptible species, and zoonotic potential. MIDV, recombinant virus originating from Semliki Forest virus and Mayaro virus (*14*), forms its own complex, rather than clustering within the Semliki Forest virus complex (*6*).

Identification of MIDV and SINV as possible neurologic pathogens in horses in South Africa prompted the investigation of undiagnosed neurologic and febrile disease or sudden unexplained death in wildlife, nonequine domestic animals, and birds. The purpose of our study was to investigate the host range and association of alphaviruses from Africa with neurologic disease and death, as well as to increase knowledge on pathogenesis and the zoonotic potential of these 2 viruses.

## Materials and Methods

### Clinical Infections

This study forms part of an ongoing passive surveillance study to detect zoonotic arboviruses in South Africa. A total of 608 EDTA-treated blood or postmortem specimens from animal species other than horses that had neurologic disease, acute febrile illness of unknown origin, or sudden unexpected death during February 2010–September 2018 in South Africa were submitted to the Centre for Viral Zoonoses, Department of Medical Virology, University of Pretoria (Pretoria, South Africa). Clinical cases were submitted by wildlife veterinarians and pathologists who identified cases that fit the case definition of febrile or neurologic signs or sudden unexpected deaths of unknown origin. EDTA-treated blood or serum samples were submitted when animals were alive, whereas tissues were submitted if the animal died. Preferred tissue type was brain matter or spinal cord and visceral organs, including lungs, spleen, and liver samples. The study was approved under Section 20 (no. 12/11/1/1) by the Department of Forestry and Fisheries and by the Animal Ethics Committee (no. V057–15 and H12/16).

We performed full necropsy examination at the Section of Pathology, Department of Paraclinical Sciences, Faculty of Veterinary Science, University of Pretoria, for animals that had clinically progressive quadriparesis, apparently normal mentation, and positive results for MIDV or SINV by reverse transcription PCR. We collected a wide range of organs and tissues from all wildlife cases and preserved the samples in 10% neutral buffered formalin for histologic examination. We microscopically examined routinely prepared, hematoxylin and eosin–stained (*18*) histologic sections by using a standard light microscope.

### RNA Extraction and PCR

We extracted all specimens under Biosafety Level 3 (BSL-3) conditions in the Department of Forestry and Fisheries compliant BSL-3 laboratory at the Centre for Viral Zoonoses, University of Pretoria. We extracted virus RNA from blood or body fluids by using a Viral Mini Kit (QIAGEN, https://www.qiagen.com) according to the manufacturer’s instructions and virus RNA from tissue samples by using the RNeasy Kit (QIAGEN). We analyzed all clinical specimens by using genus-specific nested real-time PCRs (*11*). We designed specific MIDV (forward ^6285^5′- GCAGCCTTTTGTCCGTCYAA-′3^6305^ and reverse ^6633^5′-GGCTTCAAGTCRTAGGTTT-3′^6614^) and SINV (forward ^6285^5′-GCAACCTTYTGCCCCGCYAA-′3^6305^ and reverse ^6633^5′-GGGACCAAATTATRCGTCT-3′^6613^) nested primers to increase the RNA-dependent RNA polymerase gene region from 198 bp to 348 bp for phylogenetic analysis by using the same conditions as in the alphavirus PCR (*11*). We designed primers on the basis of MIDV strain SAE_25/11 (GenBank accession no. KF680222) and SINV strain SA_AR86 (accession no. U38305). For differential diagnosis, we also screened all specimens for flaviviruses (*19*), equine encephalosis virus (*20*), and Shuni virus (*21*).

### Sanger Sequencing and Phylogenetic Analysis

Arbovirus PCR-positive results were confirmed by sequencing at Inqaba Biotec (https://www.inqababiotec.co.za). We analyzed sequence data by using CLC Main Workbench 8 (QIAGEN) and MEGA 6.06 software (https://www.megasoftware.net). We performed multiple sequence alignments by using MAFFT version 7 software (http://mafft.cbrc.jp) with default parameters and used them to assemble sequences. We conducted maximum-likelihood analysis by using RaxML (*22*) and invoking the auto-MRE bootstopping function by applying a generalized time-reversible model with gamma distribution of rates across sites. We calculated bootstrap support values by using the autoMRE bootstopping criterion in RaxML. We conducted P-distance analysis in MEGA 6.06 and determined average within mean group distance between MIDV and SINV strains. We submitted sequences >200 bp to GenBank.

### Virus Isolation

We subjected all PCR-positive samples to virus isolation on African green monkey kidney cells (Vero) and baby hamster kidney cells (BHK-21 clone 3). We maintained cells in Earle minimum essential medium (EMEM) containing l-glutamine (Lonza, https://www.lonza.com), MycoZap CL-Plus (Lonza), 10% fetal calf serum (FCS) (Lonza) for Vero or 20% FCS for BHK-21 clone 3 cells in an Intercool Incubator (Lasec, https://www.lasec.com) at 37°C and an atmosphere of 5.0% CO_2_. 

We performed isolations by using EMEM containing 10% fetal calf serum and Mycozap CL-Plus for Vero cells and 20% fetal calf serum and Mycozap CL-Plus for BHK-21 clone 3 cells and incubated at 37°C in an atmosphere of 5% CO_2_. We inoculated blood or serum samples (200 μL) from animals directly onto phosphate-buffered saline (PBS)–washed cells, incubated for 1 h, and washed once with PBS, then added 2% EMEM. We cut tissue specimens into pieces (≈5 mg) and homogenized with sterile glass beads (Merck, https://www.merck.com) at 100 × *g* for 6 min by using a TissueLyzer (QIAGEN). We then centrifuged homogenates at 1,000 × *g* for 10 min to collect debris. We used 200 μL of supernatant to infect PBS-washed cells, incubated them for 1 h, and then added 2% EMEM. We passaged cultures 3–4 times at 7-day intervals, observing monolayers daily for cytopathic effect. Between passages, we froze cultures at –80°C, thawed 3 times, and clarified by centrifuging at 1,000 × *g* for 5 min.

### Data Analysis

We performed data and statistical analyses by using Epi Info version 7.2.0.1 (https://www.cdc.gov/epiinfo/index.html) and a Fisher exact test with 95% CIs and odds ratios (ORs) to calculate the association between clinical signs and infection. We excluded animals that were found dead (n = 76) or were aborted (n = 23) or stillborn (n = 13) from OR analysis.

## Results

During the 9-year study period, we tested 608 animals that had unsolved neurologic, febrile, and respiratory signs or sudden unexpected death. We detected MIDV in 23 (3.8%, 95% CI 2.4%–5.5%) animals and SINV in 9 (1.5%, 95% CI 0.5%–2.4%) (Table 1). We detected MIDV in wildlife (16/361; 4.4%, 95% CI 2.3%–6.6%), domestic animals (5/196; 2.6%, 95% CI 0.3%–4.8%), and wild birds (2/51; 3.9%, 95% CI 0%–9.3%) and SINV in wildlife (7/608; 1.1%, 95% CI 0.5%–3.4%) and domestic animals (2/196; 1%, 95% CI 0%–2.4%) (Table 1). We did not detect SINV in clinical samples from birds.

**Table 1:** Samples from wildlife, nonequine domestic animals, and birds tested for alphavirus by using nested real-time PCRs specific for MIDV and SINV, South Africa*

Animal	No. tested	No. positive (%, 95% CI)	
		MIDV	SINV
Buffalo (*Syncerus caffra*)	54	2 (3.7, 0.0–8.7)	1 (1.9, 0.0–5.4)
Avian†	51	2 (3.9, 0.0–9.2)	0
Sable antelope (*Hippotragus niger*)	53	2 (3.8, 0.0–8.9)	2 (3.8, 0.0–8.9)
Warthog (*Phaecocherus africanus*)	26	2 (7.7, 0.0–18.0)	0
White rhinoceros (*Ceratotherium simum*)	65	6 (9.2, 2.2–16.3)	1 (1.5, 0.0–4.5)
Lion (*Panthera leo*)	9	2 (22.2, 0.0–49.4)	0
Waterbuck (*Kobus ellipsiprymnus*)	3	1 (33.3, 0.0–86.7)	0
Genet (*Genetta genetta*)	2	1 (50.0, 0.0–119.3)	1 (50.0, 0.0–119.3)
Giraffe (*Giraffa camelopardalis*)	6	0	1 (16.7, 0.0–46.5)
Blesbuck (*Damaliscus pygargus phillipsi*)	4	0	1 (25.0, 0.0–67.4)
Crocodile (*Crocodylus niloticus*)	12	0	0
Springbok (*Antidorcas marsupialis*)	4	0	0
Roan antelope (*Hippotragus equinus*)	3	0	0
Other antelope‡	82	0	0
Elephant (*Loxodonta africana*)	6	0	0
Equine (zebra/donkeys)§	10	0	0
Carnivores¶	17	0	0
Alpaca	8	0	0
Domestic bovid	93	5 (5.4, 0.8–10.0)	0
Domestic sheep	45	0	1 (2.2, 0.0–6.5)
Domestic and other porcine	5	0	1 (20.0, 0.0–46.5)
Camel	8	0	0
Goat	1	0	0
Wildlife	361	16 (4.4, 2.3–6.6)	7 (1.9, 0.5–3.4)
Domestic	196	5 (2.6, 0.3–4.8)	2 (1.0, 0.0–2.4)
Avian	51	2 (3.9, 0.0–9.2)	0
Total	608	23 (3.8, 2.4–5.5)	9 (1.5, 0.5–2.4)

*MIDV, Middelburg virus; SINV, Sindbis virus.  †Avian MIDV-positive: laughing dove (*Spilopelia senegalensis*) and blue crane (*Grus paradisea*).  ‡Kudu, wildebeest, impala.  §Zebra and donkeys.  ¶Jackal, hyena, wild dog, civet.

The 608 animals tested were from 99 animal species, of which 14 species were positive for MIDV or SINV (Table 1). We detected MIDV in white rhinoceros (9.2%, 95% CI 2.2%–16.3%), buffalo (3.7%, 95% CI 0%–8.7%), domestic bovids (5.4%, 95% CI 0.8%–10.0%), warthogs (7.7%, 95% CI 0%–18.0%), lions (22.2%, 95% CI 0%–49.4%), birds (lemon dove and blue crane; 3.9%, 95% CI 0%–9.2%), sable antelopes (3.8%, 95% CI 0%–8.9%), waterbucks (33.3%, 95% CI 0%–86.7%), and genets (50%, 95% CI 0%–119.3%) (Table 1). SINV was detected in buffalo (1.9%, 95% CI 0%–5.4%), sable antelopes (3.8%, 95% CI 0%–8.9%), rhinoceroses (1.5%, 95% CI 0%–4.5%), giraffes (16.7%, 95% CI 0%–46.5), European wild boar (16.7%, 95% CI 0%–46.5%), sheep (2.2%, 95% CI 0%–6.5%), blesbucks (25%, 95% CI 0%–67.4%), and genets (50%, 95% CI 0%–119.3%) (Table 1). One co-infection with MIDV and SINV was reported in a genet (Table 1). Two white rhinoceroses had co-infections (MVA07/10 with MIDV and Shuni virus, MVA11/10 with MIDV and equine encephalosis virus). Two animals, a domestic bovid (ZRU176/14/2) and a buffalo (ZRU160/18), had co-infections with MIDV and West Nile virus.

All SINV-positive animals and 20 (87%) of 23 MIDV PCR-positive animals had virus detected in postmortem specimens (Table 2). MIDV was detected primarily in the central nervous system (CNS; 14/23, 60.9%), followed by visceral organs (10/23, 43.5%), blood (5/23, 21.7%), and respiratory organs (2/23, 8.7%). SINV was detected primarily in the CNS (5/9, 55.6%), visceral organs (3/9, 33.3%), respiratory organs (2/9, 22.2%) and cerebrospinal fluid (1/9, 11.1%). All clinically sick animals infected with MIDV (22/22) (p<0.06) and SINV (6/6) (p=1) had neurologic manifestations as a primary diagnostic sign (Table 2). Tongue paralysis (OR 32.5, 95% CI 2.9–368.3) was associated with SINV-positive animals (p<0.05) (Table 2). Three animals were found dead and subsequently found to be positive for MIDV (waterbuck) and SINV (buffalo and blesbuck), and an aborted Merino sheep fetus was positive for SINV (Table 2).

**Table 2:** Clinical signs associated with MIDV and SINV infections in wildlife, nonequine domestic animals, and birds, South Africa*

Virus, sign, and outcome	No. (%) positive, n = 22	No (%) negative, n = 474	Odds ratio (95% CI)	p value
MIDV				
Sign				
Fever	4 (18.2)	42 (8.9)	2.3 (0.7–7.1)	0.1
Neurologic signs	22 (100.0)	404 (85.2)	ND	0.06
Ataxia	4 (18.2)	100 (21.1)	0.8 (0.2–2.5)	1.0
Paralysis	4 (18.2)	60 (12.7)	1.5 (0.5–4.7)	0.7
Quadriparesis	6 (27.3)	114 (24.1)	1.8 (0.5–3.1)	0.8
Tongue paralysis	0	4 (0.8)	ND	1.0
Recumbency	4 (18.2)	101 (21.3)	0.8 (0.3–2.5)	1.0
Dyspnea	0	81 (17.1)	ND	1.0
Hemorrhage	0	11 (2.3)	ND	1.0
Congenital deformities	0	7 (1.5)	ND	1.0
Blindness	0	11 (2.3)	ND	1.0
Icterus	0	2 (0.4)	ND	1.0
Seizure	0	29 (6.1)	ND	1.0
Outcome	n = 23	n = 585		
Sudden unexpected death	1 (4.4)	75 (12.8)	0.3 (0.0–2.3)	0.3
Abortion	0	23 (4.1)	ND	1.0
Stillbirth	0	13 (2.7)	ND	1.0
Fatal	20 (87.0)	501 (85.6)	1.1 (0.3–3.9)	1.0
SINV	n = 6	n = 490		
Sign				
Fever	1 (16.7)	45 (9.2)	2.0 (0.2–17.3)	0.5
Neurologic signs	6 (100.0)	420 (85.7)	ND	1.0
Ataxia	2 (33.3)	102 (20.8)	1.9 (0.3–10.5)	0.6
Paralysis	1 (16.7)	63 (12.9)	1.4 (0.2–11.8)	0.7
Quadriparesis	0	120 (24.5)	ND	1.0
Tongue paralysis	1 (16.7)	3 (0.6)	32.5 (2.9–368.3)	<0.05
Recumbency	2 (33.3)	103 (21.0)	1.9 (0.3–10.4)	0.6
Dyspnea	1 (16.7)	80 (16.3)	1.0 (0.1–89)	1.0
Hemorrhage	0	11 (2.2)	ND	1.0
Congenital deformities	0	7 (1.4)	ND	1.0
Blindness	0	11 (2.4)	ND	1.0
Icterus	0	2 (0.4)	ND	1.0
Seizure	0	29 (5.9)	ND	1.0
Outcome	n = 9	n = 599		
Sudden unexpected death	2 (22.2)	74 (12.7)	0.5 (0.1–2.4)	0.3
Abortion	1 (11.1)	22 (3.8)	3.3 (0.4–27.4)	0.3
Stillbirth	0	13 (2.7)	ND	0.8
Fatal	9 (100.0)	512 (85.5)	ND	1.0

*ND, not determined; MIDV, Middelburg virus; SINV, Sindbis virus.

**Figure 1:**
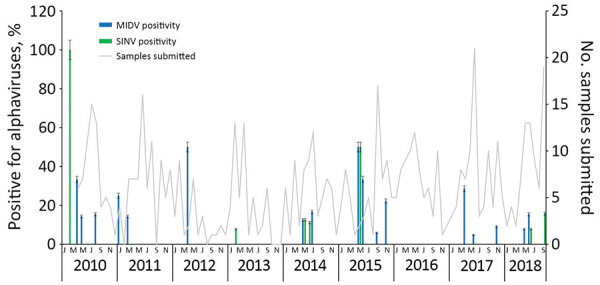
Seasonal detection of 32 alphavirus-positive infections of wildlife, nonequine domestic animals and birds, South Africa, February 2010–September 2018. Error bars indicate 95% CIs. MIDV, Middelburg virus; SINV, Sindbis virus.

**Figure 2:**
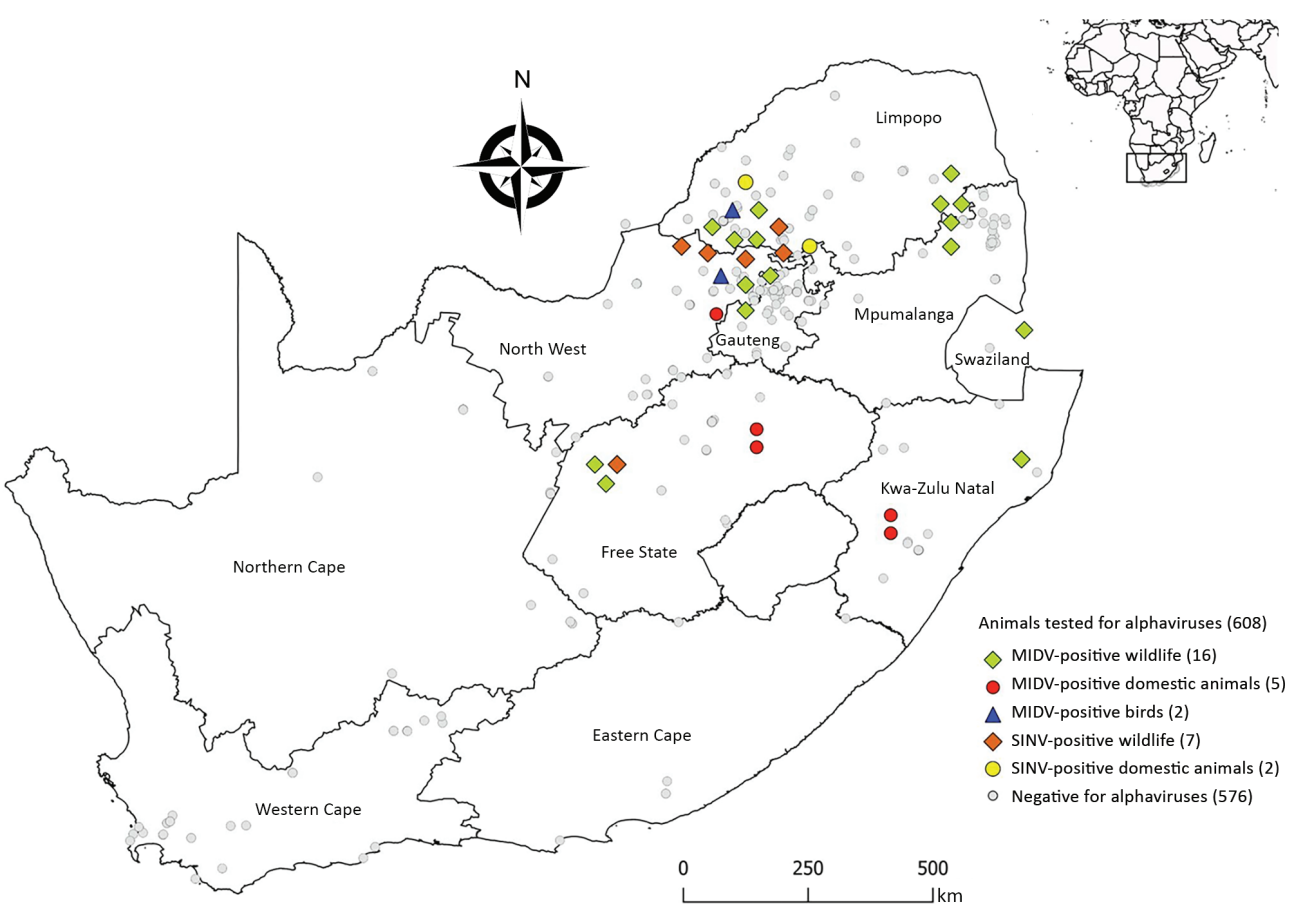
Locations of MIDV and SINV PCR-positive and –negative samples from wildlife, nonequid domestic animals, and avian species, South Africa, 2010–2018. Inset shows location of South Africa in Africa. Values in parentheses are number of animals. MIDV, Middelburg virus; SINV, Sindbis virus.

Most MIDV infections were reported during 2010 (5/62, 8.1%) and 2015 (5/60, 8.3%), followed by 2017 (4/90, 4.4%), 2014 (3/69, 4.3%), 2018 (3/75, 4.0%), 2012 (1/30, 3.3%), and 2011 (2/80, 2.5%). No MIDV infections were detected during 2016 (Figure 1). SINV infections were highest in 2018 (4/75, 5.3%), followed by 2014 (2/69, 2.9%), 2013 (1/50, 2%), and 2010 and 2015 (each 1/67, 1.5%). No SINV infections were reported during 2011, 2012, 2016, and 2017 (Figure 1). MIDV positivity was highest in April (5/55, 9.1%), followed by November (3/38, 7.9%) (Figure 1). No positive animals were reported during February, October, or December. SINV positivity was highest in September (3/65, 4.6%), followed by February (2/48, 4.2%) (Figure 1). Samples were received from all 9 provinces, although positive samples were detected in only 6 provinces (Figure 2).

No specific macroscopic lesions could be demonstrated at necropsy for cases submitted to the Section of Pathology, Faculty of Veterinary Science, University of Pretoria. Similar to other parenchymal tissues examined, no distinct cytologic nor architectural abnormalities could be demonstrated in the CNS of examined cases. Some of these animals died after a short period (<3 days) of recumbency. In these cases, secondary factors, such as dehydration and unrelenting high levels of stress, were suspected to contribute to death. In some instances, animals with severe cases were euthanized for humane reasons or as part of disease management for elective necropsy to determine the cause of outbreaks of neurologic signs in wildlife.

**Figure 3:**
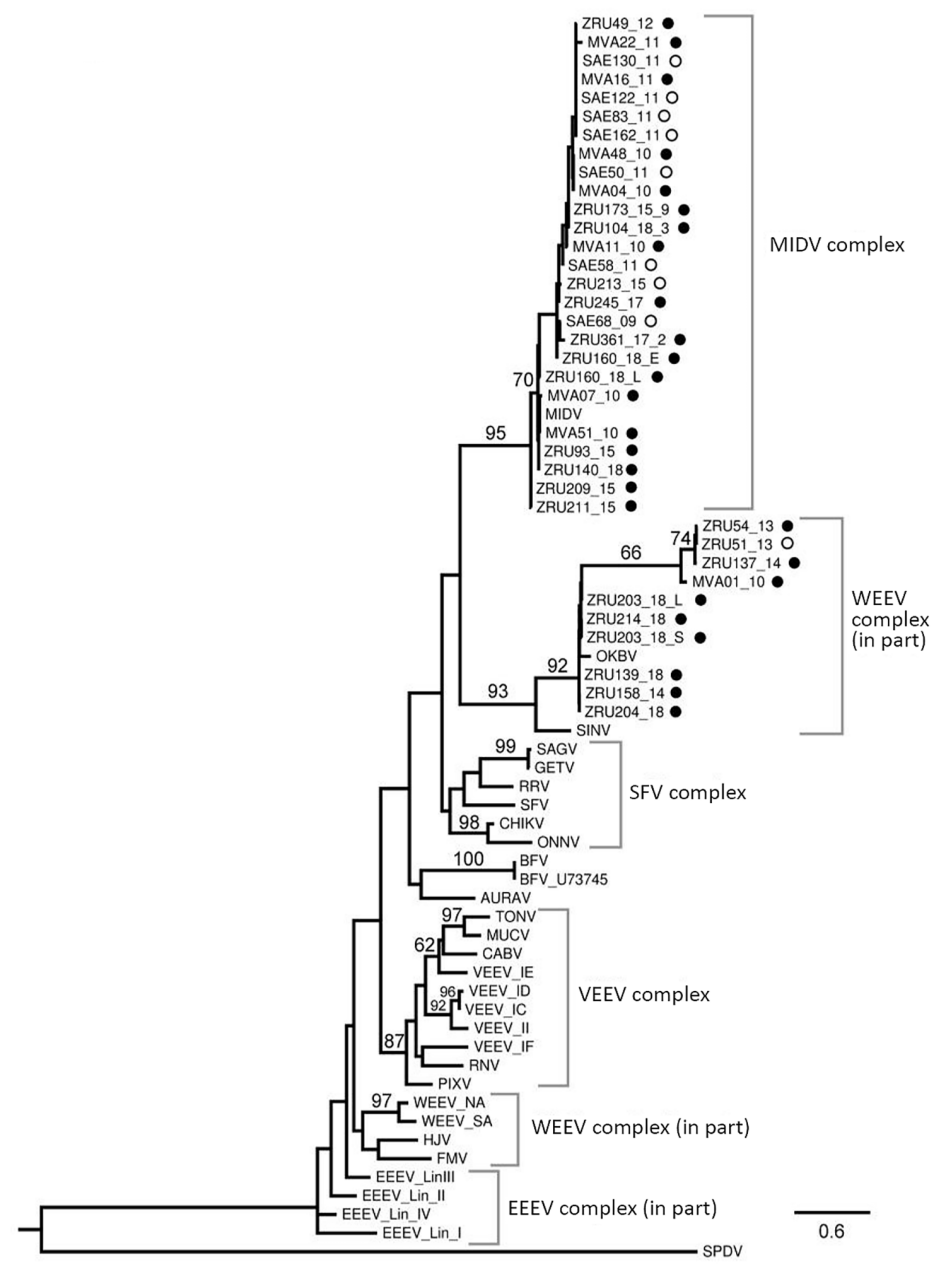
Phylogram of the RNA-dependent RNA polymerase gene (348-bp fragment) of alphaviruses rooted at the midpoint and created by using maximum-likelihood analysis (67 taxa, generalized time-reversible model with gamma distribution of rates across sites). Black circles indicate wildlife, domestic animals, and birds from South Africa, February 2010–September 2018, and open circles indicate previously reported virus-positive horses (*11*). Numbers on branches are bootstrap support values. Values are shown if they are >60. Sample identification and GenBank Accession numbers: MVA51/10, MK114099; ZRU139/18, MK114091; ZRU140/18, MK114087; ZRU158/14, MK114089; ZRU160/18, MK114092, ZRU203/18_Lung, MK114094; ZRU203/18_Spleen, MK114093; ZRU204/18, MK114095; ZRU209/15, MK114096; ZRU211/15, MK114097; ZRU214/18, MK114098; ZRU54/13, MK114090; ZRU93/15, MK114088. Reference strain, name, accession number, and origin are as described by Forrester et al. (*6*). Scale bar indicates nucleotide substitutions per site. EEEV, Eastern equine encephalitis virus; MIDV, Middelburg virus; SFV, Semliki Forest virus; SPDV, salmon pancreas disease virus; WEEV, Western equine encephalitis virus.

Phylogenetic analysis of the partial nonstructural protein 4 (nsP4) gene region (348 bp) confirmed all virus-positives cases as being infected with MIDV or SINV (Figure 3). Maximum-likelihood analysis resulted in a topology lacking strong support in deeper nodes for several alphavirus groups. However, we obtained bootstrapping values of 95 for MIDV clades and 93 for SINV clades (Figure 3). The MIDV complex had 2 separate clades within the group, and viruses detected in lions (Carnivora) were sister clades to viruses detected in rhinoceroses, warthogs, buffalo, sable antelopes, domestic bovids, and blue cranes, which clustered together (bootstrap value = 70) (Figure 3) and had a between-group mean distance of 96%. The SINV clade (Western equine encephalitis virus complex) also formed a separate cluster with the positive samples primarily from the 2018 group that was separate from positive samples reported in 2010, 2013, and 2014 (bootstrap value = 66) (Figure 3) and had a between group mean distance of 93.7%. Within-group mean nucleotide distances were 98.4% for MIDV and 95.4% for SINV.

## Discussion

We identified a total of 32 alphavirus infections in wildlife, nonequine domestic animals, and 2 birds that had neurologic or febrile signs or unexplained death over a period of 9 years (2010–2018) as MIDV (n = 23) or SINV (n = 9). Detection of these viruses in the CNS indicates that they are able to cross the blood–brain barrier and suggests that they might cause pathologic changes, neurologic disease, and death in infected animals. Detection of viral RNA in respiratory organs and visceral organs suggest spread of these viruses throughout the body. The success rate of virus isolation from neural tissue is low because death is often the end stage of disease concurrent with a low virus titer, which can often only be detected by nested PCR. Virus isolation can also be related to the quality of clinical specimens received from wildlife and domestic animals, which were often found dead in remote areas and took some time to reach the laboratory, as compared with equine cases, which are often detected earlier by owners during the stage of clinical disease and therefore are sampled earlier.

All cases were accompanied by a case investigation form with clinical and epidemiologic data recorded by the submitting veterinarian. All animals that had clinical information available had neurologic signs suggesting infection with MIDV and SINV as likely etiologies of neurologic disease (p = 0.06) despite the small sample size of alphavirus-positive cases compared with alphavirus-negative cases. None of the other signs could clearly be associated with these viruses because of the small sample size of virus-positive animals. This finding suggests that these Old World alphaviruses might have similar characteristics to their New World relatives in some species (*11*,*23*). SINV was strongly associated with tongue paralysis, as observed in the giraffe (ZRU54_13). Although the overall positivity rate was low for samples tested during this study, specific species were positive more frequently for alphaviruses. These species include white rhinoceros, buffalo, and sable antelope, all of which had >1 infections for MIDV and SINV.

MIDV was reported more frequently in white rhinoceroses and domestic bovids. SINV was not detected in domestic bovids but was detected in sheep and domestic porcines. In a few instances, samples from an animal were submitted from a cluster of animal deaths and apparent outbreak scenarios. A MIDV-positive white rhinoceros (MVA004/10) was given a diagnosis after 9 rhinoceroses from the same population had similar clinical signs. All animals were subjected to postmortem investigation over a 2-year duration at the Faculty of Veterinary Science, University of Pretoria. CNS tissues from an adult white rhinoceros (MVA007/10) from Broederstroom, North West Province, showed positive results for MIDV and equine encephalosis virus. A rhinoceros calf that was kept in the same boma (livestock enclosure) had similar signs and had died 5 days earlier. A warthog (MVA51/10) was part of a disease outbreak involving ≈50 similar cases in contact animals of the same species in Marekele National Park, Limpopo Province. All warthogs of the specific sounder (group) showed signs of ataxia that lasted 3–7 days, after which some recovered and some died. A captive-bred African buffalo calf from Bloemfontein, Free State Province, also died after paralysis developed, and an uncountable number of animals in the herd were positive for MIDV. These 3 cases of disease can be regarded as disease outbreaks affecting multiple contact animals from the same epizootic unit.

Phylogenetic analyses based on the partial nsP4 gene support the monophyletic grouping of SINV with the New World viruses in the Western equine encephalitis virus complex (*24*,*25*). The analysis also supports the grouping of MIDV strains into a genetic complex, with a 95% bootstrap support (*23*), with MIDV sequences obtained from 2 lions (ZRU209_15 and ZRU211_15), forming a sister group to the other virus-positive animals. A mean nucleotide distance of 96% was observed between the 2 groups. The 2 MIDV-positive lions originated from Mpumalanga Province, and the rest of the MIDV-positive animals were from Gauteng, Limpopo, Free State, Northern Cape, Western Cape, and KwaZulu-Natal Provinces. This clade groups with MIDV strains previously identified in horses from these areas (*11*). These findings could indicate geographic clustering between strains.

Similar results were obtained for the SINV strains, although positive samples clustered according to year, with a between group mean nucleotide distance of 93.7%, possibly indicating changes in the strains circulating over time. Because birds are believed to be reservoir hosts for SINV, new strains might be introduced through migratory birds. P-distance analysis showed little or no nucleotide variation in the nsP4 gene region for MIDV and SINV strains respectively identified in this study, apart from the limited differences described above, indicating genetically similar strains. However, this gene segment is highly conserved, and sequence lengths were relatively short (198–348 bp). Attempts to amplify more gene regions, as well as virus isolation, were not successful.

The results of this study demonstrate that alphaviruses might be associated with neurologic disease in wildlife, nonequine domestic animals, and birds in South Africa. Wide geographic distribution of MIDV and SINV in Africa suggests that this distribution should be investigated in other regions. Humans living near wildlife and livestock, such as domestic bovids, sheep, and porcines, might have similar exposure to mosquito vectors and should therefore also be monitored for potential zoonotic infections. SINV has been shown to have birds as its reservoir host and is a well-known human pathogen in South Africa, although most described cases are nonneurologic and associated with febrile disease or arthralgia. Our study identified SINV in neural tissue of various wildlife and nonequine domestic species that had neurologic signs of infection. The geographic range, prevalence of infection in various species, species-specific differences, and seroprevalence need to be determined to define the epidemiology of these pathogens. Serologic evidence for MIDV in humans has been demonstrated in South Africa (*26*), although such evidence is less informative for SINV. This finding might be caused by host range preference and spread of the vectors associated with these viruses.

Reservoir hosts for MIDV are unknown. Our suggestion of a higher prevalence of MIDV relative to SINV warrants further investigation. Widespread distribution of MIDV suggest birds might play a role in the spread of this virus. However, a dove and blue crane were the only virus-positive birds identified, both submitted as postmortem specimens. Further investigation is needed regarding the potential of the identified species to function as reservoir and amplification hosts and potential for transmission of virus to vectors. Spillover of MIDV into humans through adaption to additional vectors should be monitored. Investigation of neurologic cases and deaths in wildlife and domestic animals suggested that most cases occur during January–July—from the second half of the summer months to late autumn or early winter in warmer parts of the country, where many wildlife reside—after which vector numbers usually decrease because of colder winter temperatures.

Future studies into the epidemiology of SINV and especially MIDV would greatly benefit from analysis of increased sample sizes or whole-genome sequencing, combined with serologic analyses of affected species, especially in outbreak investigations. Syndromic surveillance for febrile and neurologic disease in sensitive species might act as an early warning system for outbreaks of emerging and reemerging arboviruses and predict outbreaks in humans (*27*,*28*).

A limitation of our study is that all other possible infectious and noninfectious etiologies could not be excluded by comprehensive investigations for all cases, suggesting that the causative link with clinical signs still has to be regarded with caution. Also, serum samples from PCR-positive animals were not available because most animals were tested postmortem, which limited validation by serologic assays for this study. However, use of serum samples should be a focus of future investigations.

Most emerging zoonotic diseases are believed to be caused by pathogens that originate from wildlife (*29*). Wildlife might either assume the role of reservoir or amplification host with or without clinical disease or be dead end or incidental hosts that might have severe disease (*30*,*31*). To our knowledge, MIDV and SINV neurologic infections have not been reported in wildlife and nonequine domestic animals. Our study demonstrated a wide host range for these viruses, and detection of these viruses directly in the neurologic tissue in several cases suggests crossing of the blood–brain barrier and MIDV and SINV as the probable cause of neurologic signs. This finding highlights the need for surveillance of alphaviruses to prevent spillover events and outbreaks in humans.
